# Advances in Human and Animal Germinal Center Models In Vitro: New Approaches to Studying Immune Responses

**DOI:** 10.1155/bmri/2510652

**Published:** 2026-10-05

**Authors:** Rocio Quinto-Manzanares, Alondra Yareli Martínez-Mora, Velia Verónica Rangel-Ramírez, Jesús Martínez-Barnetche, Hilda Minerva González-Sánchez

**Affiliations:** ^1^ Centro de Investigación Sobre Enfermedades Infecciosas Instituto Nacional de Salud Pública Cuernavaca Morelos Mexico insp.mx; ^2^ Instituto de Investigación en Ciencias Básicas y Aplicadas Universidad Autónoma del Estado de Morelos Cuernavaca Morelos Mexico uaem.mx; ^3^ Facultad de Medicina Universidad Autónoma del Estado de Morelos Cuernavaca Morelos Mexico uaem.mx; ^4^ Centro de Biociencias Universidad Autónoma de San Luis Potosí San Luis Potosí Mexico uaslp.mx; ^5^ Secretaría de Ciencia Humanidades Tecnología e Innovación SECIHTI IxM Mexico City Mexico

**Keywords:** B cells, germinal centers, in vitro models, memory B cells, plasma cells

## Abstract

Germinal centers (GCs) are dynamic structures in secondary lymphoid organs where B cells undergo antigen‐driven clonal selection, affinity maturation by somatic hypermutation (SHM), and class switch recombination (CSR), resulting in the generation of high‐affinity antigen‐specific long‐lived plasma cells and memory B cells. Reconstructing these processes in vitro is critical for mechanistic studies and for advancing translational applications in vaccinology and immunotherapy. This review examines human and murine approaches to in vitro models of GC‐like reactions, including conventional cultures, three‐dimensional (3D) scaffolds, organoids, and microfluidic systems. We summarize the stimuli and culture conditions used across studies, highlighting common requirements, as well as model‐specific strategies. Most models effectively induced B cell proliferation, CSR, and plasma cell differentiation. In contrast, SHM and clonal selection, key features of the GC, were less frequently shown, reflecting the technical complexity of reproducing these events in vitro. Finally, we outline the main challenges in recreating a fully functional GC and suggest future directions for refining these models to more closely mimic the secondary lymphoid organ environment.

## 1. Introduction

A central aspect of the adaptive humoral immune response is its ability to produce high‐affinity antibodies through iterative cycles of somatic hypermutation (SHM) and affinity‐based clonal selection [1–3]. Upon pathogen entry, antigen is captured and processed by antigen‐presenting cells, initiating adaptive immune responses through activation of cognate CD4^+^ T cells and B cells [1, 2]. Their subsequent interaction within secondary lymphoid organs promotes the formation of germinal centers (GCs), transient specialized microanatomical sites of T cell‐dependent B cell clonal expansion, selection, and differentiation [1–3].

In GCs, B cells undergo SHM of their immunoglobulin variable region genes, generating intraclonal diversity. This is followed by affinity maturation, a Darwinian‐like selection process that favors B cell clones with enhanced antigen‐binding capacity [4]. GCs are divided into a dark zone (DZ), where B cells, called centroblasts, proliferate and mutate their B cell receptors (BCRs), and a light zone (LZ), where these clones, now centrocytes, interact with antigen displayed on follicular dendritic cells (FDCs) and follicular helper T cells (Tfh) to undergo affinity‐based selection [1, 3]. Upon major histocompatibility complex class II (MHC‐II) antigen presentation by selected B cells, Tfh cells provide essential costimulatory signals and cytokines that drive B cell survival, proliferation, and differentiation [5]. Positively selected B cells either re‐enter the DZ to receive additional cycles of SHM or exit the GC to differentiate into long‐lived plasma cells, capable of secreting high‐affinity antibodies, or memory B cells, which provide rapid and robust responses upon re‐encounter with antigen [3]. This highly dynamic process is crucial not only for the generation of effective humoral immunity but also for establishing long‐term immune memory, a defining feature of immune protection and successful vaccination [1–3].

Despite extensive research, several key aspects of GC biology remain unresolved, including the molecular and cellular mechanisms that determine GC termination, the rules of clonal competition in response to complex, multiepitope antigens, and the processes by which B cells measure their affinity [2, 6, 7]. These knowledge gaps have motivated the establishment of alternative experimental systems that can reproduce GC‐like dynamics under controlled conditions. While murine models have provided fundamental insights into GC biology, important interspecies differences limit the direct extrapolation of these findings to human immune responses [1, 2, 8]. Therefore, the development of human in vitro GC models has become increasingly important to complement mechanistic studies in mice and to provide experimental platforms with greater physiological relevance for studying human immunity [9–11].

In recent years, several in vitro models have been created to mimic the key biological processes of GCs [9–12]. Such systems aim to provide a controlled and reproducible alternative to in vivo studies, enabling a clearer understanding of GC biology and B cell differentiation [9–11]. Recent strategies include cocultures, three‐dimensional (3D) cultures, lymphoid organ fragments, and microfluidic devices [9–13]. Although these approaches offer valuable experimental platforms, their ability to replicate the full complexity and dynamic nature of GCs is still being refined.

These platforms hold significant potential to advance vaccine design, evaluate personalized immunotherapies by testing drug or vaccine efficacy under diverse clinical or immunological conditions using patient‐derived organoids, and provide deeper insights into human humoral immunity [9–11]. This review summarizes current in vitro GC models, with a particular emphasis on B cell biology, integrating findings from both human and mouse studies. We compare the methods used to replicate GC‐associated processes, highlighting the roles of cytokines and cell–cell interactions in B cell activation, proliferation, and differentiation. Our goal is to synthesize current knowledge, identify the limitations of existing platforms, and highlight opportunities to refine them for a more faithful representation of human GC biology.

## 2. Search Strategy and Selection Criteria

The objective of this review was to gather information on protocols used to recreate in vitro models of GC responses in human and mouse systems. Due to the limited data available, we broadened our search to include studies where B cells were stimulated in vitro, focusing on the GC‐related processes observed in these protocols. Our research question was: “What in vitro models and B cell stimulation protocols have been developed to recapitulate key aspects of GC responses in human and mouse systems?”

We searched the PubMed database considering studies published up to May 28, 2026. Search terms included “Germinal center AND B cells AND (in vitro OR ex vivo OR organoids OR culture OR stimulation)” and “Memory B cells AND (plasma OR antibody‐producing cells) AND (in vitro OR ex vivo OR organoids OR culture OR stimulation).” Retrieved studies were screened and categorized by the biological system used. Mouse‐based and human‐based models were analyzed separately and compared.

Two review authors independently conducted the search and selected studies in two phases. First, articles that clearly did not meet inclusion criteria based on title and abstract were excluded, including duplicates and review articles. Next, the full texts of selected articles were screened, and studies not fulfilling inclusion criteria were excluded. Full texts that were unavailable and preprints were also excluded. Disagreements were resolved through discussion or by a third reviewer if necessary. The study selection process is summarized in Figure 1.

**Figure 1:**
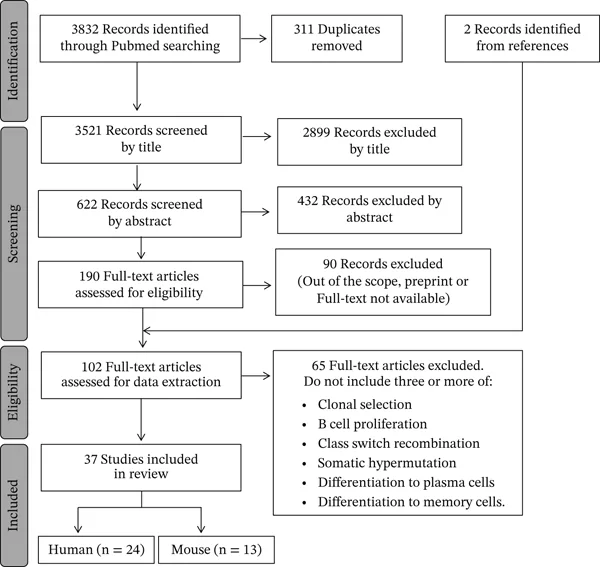
Flow diagram of the article selection process.

Studies were eligible if they included in vitro culture and stimulation of B cells from peripheral blood, tonsils, or other lymphoid organs. B cells could be naïve, memory, or total B cells, cultured alone or in coculture with other cell types. All culture systems were considered, including conventional cultures, 3D models, and microfluidic platforms.

For comparative analysis, we selected studies assessing three or more key GC‐associated processes: clonal selection, B cell proliferation, class switch recombination (CSR), SHM and affinity maturation, plasma cell differentiation, and memory B cell differentiation. Clonal selection was identified by experiments involving stimulation with antigens with or without repertoire sequencing to analyze the expansion of specific clones; studies that used polyclonal activators such as anti‐BCR or CpG were considered not to allow clonal selection. B cell proliferation was recognized in studies that measured increases in total B cell numbers or analyzed DNA synthesis or cell division markers. CSR assessment included analysis of immunoglobulin isotypes, germline and postswitch transcripts, as well as switch circle transcripts indicative of ongoing recombination events. SHM evaluation required sequencing of heavy or light chain genes (IGH or IGL), or expression of genes involved in SHM, such as activation‐induced cytidine deaminase (AID). Plasma cell differentiation was recognized when studies measured phenotypic markers like CD138 or CD38 [14], quantified antibody‐producing cells, or examined gene expression of plasmablast/plasma cell markers such as BLIMP‐1 (PRDM1) and XBP1 [15]. Memory B cell differentiation was assessed by phenotyping analysis with markers such as CD27 or by a secondary response test in vivo.

For each eligible study, data were extracted on the biological source, culture model, stimulation strategy, coculture conditions, and GC‐associated processes evaluated. Studies were categorized according to their primary focus (GC biology, immunological memory, or antibody production). Studies evaluating three or more GC‐associated processes were selected for detailed comparative analysis.

## 3. Synthesis of Results

A total of 3832 articles were identified through the database search, with 311 duplicates removed. After title screening, 622 articles were selected, of which 190 remained after abstract evaluation. Full‐text review reduced this number to 100. Two additional relevant articles were identified through references of the reviewed full texts. Based on their primary focus, the included studies were classified as investigating GC biology, immunological memory, or antibody production (Tables S1 and S2).

Among the included studies, those evaluating at least three GC‐associated processes were selected for detailed comparative analysis. This subset included 24 studies for human B cells and 13 for mouse models, summarized in Tables 1 and 2. Among human studies, three described all searched characteristics [9–11], whereas two did not assess antigen‐driven clonal selection [16, 17] and one lacked evidence of SHM [12]. For mouse models, two fulfilled all criteria [35, 36], whereas one met all except SHM [37].

**Table 1:** Description of studies analyzing key germinal center processes using human B cells in vitro. Analyzed characteristics include clonal selection, B cell proliferation, somatic hypermutation (SHM), class switch recombination (CSR), and differentiation into memory or plasma cells. The type of B cell used, the stimuli employed, and whether coculture was used in the experimental protocol are also detailed.

ID	Reference	Clonal selection	B prolif	SHM	CSR	Bmem diff	PC diff	B cell type	Stimuli	Coculture
1	Zhong, et al., 2025 [11]	∗	∗	∗	∗	∗	∗	Tonsil mononuclear cells; PBMCs	IL‐4, BAFF, IL‐21, antigen (± R848); PEG‐4MAL matrix (with adhesive peptides GFOGER + RGD), CXCL12, CD40L‐expressing cells	Coculture with CD40L‐expressing fibroblasts and human‐tonsil‐derived FDCs
2	Wagar et al., 2021 [9]	∗	∗	∗	∗	∗	∗	Tonsil mononuclear cells	Antigens, BAFF	Autologous tonsil tissue
3	Goyal et al., 2022 [10]	∗	∗	+/−	∗	∗	∗	Näive B cells	IL‐4 and anti‐CD40, antigen	Coculture with T cells and monocyte‐derived DCs
4	Braham et al., 2026 [12]	∗	∗		∗	∗	∗	Tonsil mononuclear cells	Viral antigens and vaccines, with or without R848	Tonsil‐derived fibroblastic reticular cells (FRCs), autologous or allogeneic, in 2D and 3D PEG‐4MAL hydrogels
5	Verstegen et al., 2023 [16]		∗	∗	∗	∗	∗	Näive B; memory B	IL‐4, IL‐21, and CD40L‐expressing cells	Coculture with CD40L‐expressing 3T3 cells
6	Suthanthiraraj et al. 2025 [17]		∗	∗	∗	∗	∗	Näive B cells	Microbead‐bound CD40L, anti‐BCR, anti‐CD21, CpG, BAFF, IL‐2, IL‐4, IL‐10, IL‐21	No
7	Sanjuan Nandin et al., 2017 [18]	∗	∗		∗		∗	Memory B	Particulate anti–*κ*‐CpG, IL‐15, IL‐6, antigen	No
8	Choe et al., 1997 [19]		∗		∗	∗	∗	GC B cells from tonsils	IL‐2, IL‐4, IL‐10, CD40L, anti‐Ig	HK cells (FDC‐like cell line)
9	Arpin et al., 1995 [20]		∗		∗	∗	∗	GC B cells	IL‐2, IL‐10, IL‐4, CD40L‐expressing fibroblasts	CD40L‐expressing fibroblasts
10	Huggins et al., 2007 [21]		∗		∗	∗	∗	Näive B; memory B	CpG, IL‐2, IL‐10, IL‐15, anti‐CD40, IFN*α*, HGF, HA	No
11	Braham et al., 2023 [22]		∗		∗	∗	∗	Total B cells	CpG, IL‐2, IL‐4, IL‐6, IL‐10, IL‐15, IL‐21, IFN*α*, APRIL, CD40L‐expressing fibroblasts, PEG‐4MAL matrix + RGD + GPQ‐W	CD40L‐expressing fibroblasts, human tonsil‐derived stromal cells
12	Fecteau et al., 2009 [23]		∗	∗	∗		∗	Näive B; memory B	IL‐2, IL‐4, IL‐10, and CD40L‐expressing fibroblasts	CD40L‐expressing fibroblasts
13	Unger et al., 2021 [24]		∗		∗	∗	∗	Näive B	IL‐21, IL‐4, anti‐IgM, and CD40L‐expressing fibroblasts	CD40L‐expressing fibroblasts
14	Ettinger et al., 2005 [25]		∗		∗		∗	Näive B; memory B (cord blood or PB)	IL‐2, IL‐4, IL‐21, IL‐10, anti‐CD40, anti‐IgM, or SAC	No
15	Kwakkenbos et al., 2010 [26]		∗	∗			∗	Memory B	Transduction of Bcl‐6 and Bcl‐xL, IL‐21, IL‐2, IL‐4, CD40L‐expressing fibroblasts	CD40L‐expressing fibroblasts
16	Conde et al. 2026 [27]		∗	∗			∗	Memory B	IL‐2, IL‐21, feeder cells	Irradiated NB21 feeder cells, irradiated autologous PBMCs
17	Vásquez et al., 2015 [28]		∗		∗		∗	Näive B; memory B	CpG, IL‐2, IL‐6, IL‐10	3T3 fibroblasts
18	Helm et al., 2023 [13]		∗		+/−		∗	Tonsil B cells	CD40L, IL‐21, BAFF	No
19	Koers et al., 2022 [29]		∗		∗		∗	Näive B	CD40L, IL‐21	No
20	Ding et al., 2013 [30]		∗		∗		∗	Näive B	IL‐2, IL‐4, IL‐21, supernatants from CD40L‐CD8‐transfected J558 cell line	HK cells
21	Kuchen et al., 2007 [31]		∗		∗		∗	Näive B	CD40L, IL‐21	No
22	Dubois et al., 1998 [32]		∗		∗		∗	Näive B	IL‐12	DCs (human monocyte derived)
23	Grimaitre et al., 1997 [33]		∗		∗		∗	Total B cells (cord blood or PB)	Coculture with EL‐4	EL‐4 (murine Th2 cell line)
24	Galibert et al., 1996 [34]		∗			+/−	+/−	Tonsil B cells (CD38^−^)	CD40L, anti‐BCR, anti‐CD19, and IFN*α* or IFN*γ*	CD32‐transfected or CD40L/CD32‐double transfected fibroblasts

*Note:* +/− indicates that the characteristic was partially assessed.

Abbreviations: APRIL, a proliferation‐inducing ligand; BAFF, B‐cell activating factor; BCL‐6, B‐cell lymphoma 6 protein; CD40L, CD40 ligand; CpG, cytosine‐phosphate‐guanine; CXCL12, C‐X‐C motif chemokine ligand 12; DCs, dendritic cells; FDCs, follicular dendritic cells; GC, germinal center; GPQ‐W, cross‐linker GCRD*GPQ*GIWGQDRC; HA, hyaluronic acid; HGF, hepatocyte growth factor; IFN*α*, alpha interferon; IFN*γ*, gamma interferon; IL, interleukin; PB, peripheral blood; PBMCs, peripheral blood mononuclear cells; PEG‐4MAL, four‐armed polyethylene glycol‐maleimide; RGD, Arg‐Gly‐Asp; SAC, *Staphylococcus aureus* Cowan I.

**Table 2:** Description of studies analyzing key germinal center processes using mouse B cells in vitro. Analyzed characteristics include clonal selection, B cell proliferation, somatic hypermutation (SHM), class switch recombination (CSR), and differentiation into memory or plasma cells. The type of B cell used, the stimuli employed, and whether coculture was used in the experimental protocol are also detailed.

ID	Reference	Clonal selection	B prolif	SHM	CSR	Bmem diff	PC diff	B cell type	Stimuli	Coculture
1	Moeller, et al., 2023 [35]	∗	∗	∗	∗	∗	∗	Näive B	Glyconjugate vaccine candidates, IL‐4, matrix scaffold (PEG‐4MAL hydrogel with REDV peptides), (BAFF and CD40L from 40LB cells)	40LB cells (CD40L‐ and BAFF‐expressing fibroblast)
2	Roh, et al., 2018 [36]	∗	∗	∗	∗	∗	∗	Näive B	IL‐4, BAFF, IL‐21, microbead‐bound CD40L, OVA‐coated or NP‐coated microbeads	No
3	Martínez‐Riaño, et al., 2023 [37]	∗	∗		∗	∗	∗	Näive B cells from B1‐8^hi^ (NP‐specific) mice	1‐*μ*m latex beads coated with NP‐OVA (BCR phagocytic antigen)	Coculture with OVA‐specific OT‐2 CD4^+^ T cells
4	Nojima, et al., 2011. [38]		∗		∗	∗	∗	Näive B	IL‐4, IL‐21 (BAFF and CD40L from 40LB cells)	40LB cells
5	Purwada, et al., 2019 [39]	∗	∗		∗		∗	Naïve B; GC B cells	IL‐4, anti‐IgM or NP‐OVA, anti‐Fas, (BAFF and CD40L from 40LB cells), matrix scaffold (PEG‐MAL hydrogel with REDV or RGD peptides)	40LB cells
6	Béguelin, et al., 2017 [40]		∗	∗	+/‐		∗	Naïve B; GC B cells	IL‐4, matrix scaffold (RGD‐presenting hydrogel reinforced with SiNP), (BAFF and CD40L from 40LB cells)	40LB cells
7	George A, et al., 1991 [41]		∗		∗		∗	GC B cells from Peyer′s patches	Not applied	Microculture with helper T cells and DCs.
8	Purwada A, et al., 2015 [42]		∗		∗		∗	Naïve B; GC B cells	IL‐4, matrix scaffold (RGD‐presenting hydrogel reinforced with SiNP), (BAFF and CD40L from 40LB cells)	40LB cells
9	Lopez‐Ocasio M, et al., 2020 [43]		∗		∗		∗	Näive B	Anti‐CD3, anti‐IgM, IL‐4, IL‐21	Tfr in the presence or absence of Tfh.
10	Kramer L, et al., 2022 [44]		∗		∗		∗	Näive B	IL‐4, IL‐21, microbead‐bound CD40L, BAFF, anti‐IgM loaded liposomes	No
11	Graney PL, et al., 2022 [45]	∗	∗			∗		Näive B	IL‐4, IL‐9, IL‐21, anti‐IgM or *Klebsiella* antigens, matrix scaffold (PEG‐4MAL hydrogel with REDV peptides), (BAFF and CD40L from 40LB cells)	40LB cells
12	Grund LZ, et al., 2013 [46]		∗		∗		∗	Memory B cells	Anti‐CD40, IL‐2, IL‐4, IL‐10, CpG, IL‐6 or IL‐17A, IL‐21, IL‐23 or IL‐33	No
13	Robinson & Tarlinton, 2025 [47]		∗		∗		∗	Naïve B cells	IL‐4, IL‐21, (BAFF and CD40L from 40LB cells)	40LB cells

*Note:* +/− indicates that the characteristic was partially assessed.

Abbreviations: BAFF, B‐cell activating factor; BCR, B cell receptor; CD40L, CD40 ligand; DCs, dendritic cells; GC, germinal center; IL, interleukin; NP, used 4‐hydroxy‐3‐nitrophenylacetyl hapten; OVA, ovalbumin; PEG‐MAL, polyethylene glycol‐maleimide; PEG‐4MAL, four‐armed polyethylene glycol‐maleimide; REDV, Arg‐Glu‐Asp‐Val; RGD, Arg‐Gly‐Asp; SiNP, silicate nanoparticles; Tfh, T follicular helper cells; TLR, Toll‐like receptor.

B cell proliferation was the most frequently reported characteristic across both human and mouse models, followed by plasma cell differentiation and CSR (Figure 2A,B). In contrast, SHM, a hallmark of GC function, was least frequently reported (Figure 2A,B). This likely reflects the relative ease of detecting B cell proliferation using general markers of cell division, which are commonly included even though they are not specific to GC reactions. In contrast, SHM requires more technically demanding approaches, such as repertoire sequencing or mutation analysis in immunoglobulin variable regions, which may explain its limited evaluation. Although clonal selection is critical for GC formation and function, most studies used polyclonal stimulation (anti‐BCR, superantigen). Only a few investigated antigen‐driven responses to identify reactive B cell populations [9–12, 18, 35–37, 39, 45].

**Figure 2:**
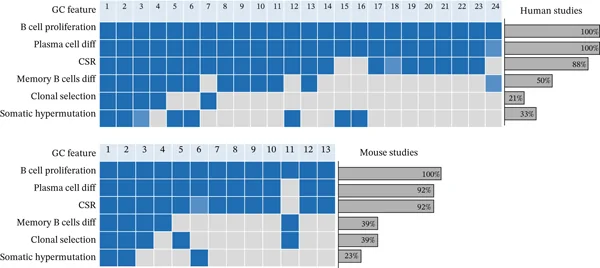
Distribution of germinal center (GC) features reported across the studies included in this review. Among the 24 human and 13 mouse models analyzed, the figure shows how many studies documented clonal selection, B‐cell proliferation, class‐switch recombination (CSR), somatic hypermutation (SHM), or differentiation into plasma and memory cells.

Both mouse and human models have evolved from simple cell cultures to 3D configurations and more complex platforms that attempt to more accurately reproduce the lymphoid microenvironment. The most recent approaches employ synthetic hydrogels, biological matrices, or organ‐on‐a‐chip microfluidic devices.

## 4. Experimental Human Models Simulating Elements of the GC Reactions

The majority of studies used naïve B cells as the starting population [10, 17, 24, 29–32], reflecting their physiological role as the primary population recruited into GCs, where they undergo clonal selection, SHM, and class switching [48]. Other studies used memory B cells [18, 26, 27], or tested both naïve and memory B cell populations [16, 21, 23, 25, 28]. Only a few studies analyzed total B cells isolated from peripheral blood [22, 33] or tonsils [13, 34]. Other populations, such as GC B cells [19, 20], tonsil mononuclear cells [9, 11, 12], and peripheral blood mononuclear cells (PBMCs) [11], were also used (Table 1). Although memory B cells are commonly used to study secondary responses and plasma cell differentiation, they have been reported to re‐enter GCs to continue their diversification [49].

Cocultures were frequently used. These included B cells cocultured with CD40L‐expressing fibroblasts [16, 20, 23, 24, 26, 34], dendritic cells [32], FDCs [19, 30], T cells [33], fibroblasts [28], tonsil mononuclear cells [9], tonsil mononuclear cells together with autologous or allogeneic tonsil‐derived fibroblastic reticular cells (FRCs) [12], CD40L‐expressing fibroblasts and FDCs [11], T cells and dendritic cells [10], CD40L‐expressing fibroblasts with tonsil‐derived stromal cells [22], and irradiated autologous PBMCs together with murine NB21 feeder cells (40LB‐derived fibroblasts expressing CD40L, BAFF, and IL‐21 [50]) [27].

Most studies used conventional cultures (Figure 3A), whereas six employed 3D culture systems [9–13, 22] (Figure 3B–G). These models include synthetic hydrogels (such as polyethylene glycol‐4 arm‐maleimide [PEG‐4MAL]) [11, 12, 22], biological matrices (Matrigel, collagen) [10], or polyelectrolyte microcapsules loaded with cytokines [13] (Figure 3C–F). Some, such as those by Zhong et al. [11] and Goyal et al. [10], integrate “organ‐on‐a‐chip” systems with microfluidic flow that allow microenvironmental control and maintenance of functional B and T cell populations (Figure 3D,F). These models represent significant progress in mimicking human GC‐like environments; however, each model still presents some limitations, such as dependence on donor availability and variability [9, 12]; lack of direct antigenic stimulation [13, 22] or cellular diversity [13]; or requirement for specialized technology and infrastructure [10, 11] (Table S3).

**Figure 3:**
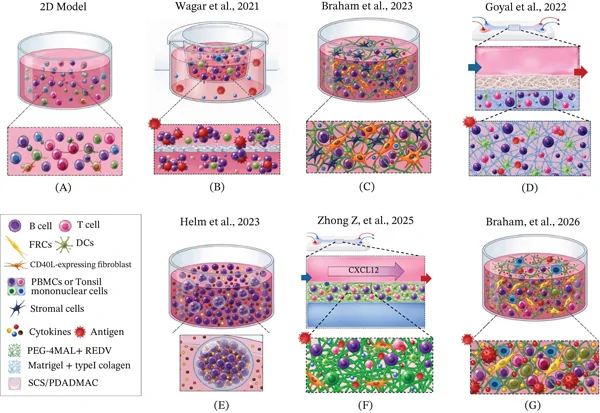
Schematic overview of 3D human culture systems used to recreate GC‐like features. (A) General layout of conventional 2D cultures. (B) Wagar et al., 2021: high‐density tonsil single‐cell suspensions cultured on permeable membranes with antigen stimulation. (C) Braham et al., 2023: PEG‐4MAL hydrogel‐based 3D matrix coculturing B cells with CD40L^+^ fibroblasts and tonsil stromal cells. (D) Goyal et al., 2022: organ‐on‐a‐chip platform with dynamic flow, Matrigel/Type I collagen ECM, B cells, T cells, and autologous dendritic cells. (E) Helm et al., 2023: encapsulation of tonsillar B cells in semipermeable microcapsules within cytokine‐supplemented medium. (F) Zhong et al., 2025: PEG‐4MAL hydrogel functionalized with REDV peptides (VCAM‐1 mimetic) supporting cocultures of tonsil mononuclear cells or PBMCs with tonsil‐derived follicular dendritic cells and CD40L^+^ fibroblasts. (G) Braham et al., 2026: PEG‐4MAL hydrogel‐based 3D matrix coculturing tonsil mononuclear cells with autologous or allogeneic FRCs.

Beyond cellular and structural components, the GC microenvironment is also defined by distinct biochemical and physical conditions that influence B cell fate. Koers et al. [29] analyzed the impact of oxygen tension on human B cell differentiation, mimicking the physiological pO₂ gradients that exist between the GC dark and LZs. They showed that oxygen levels dynamically regulate differentiation trajectories and IgG CSR, emphasizing the need to add physiologically relevant microenvironmental cues to in vitro GC models.

### 4.1. Approaches Reproducing Several Interconnected GC Features

Several studies have successfully replicated multiple GC events. Human tonsil‐derived organoids [9] (Figure 3B) and two organ‐on‐a‐chip models, one using PBMC organoids in a matrix with adhesive peptides [11] (Figure 3F) and another using Matrigel and collagen I [10] (Figure 3D), induced all evaluated processes: clonal selection, B cell proliferation, SHM, CSR, and differentiation into memory and plasma cells. Three additional studies reproduced all processes but did not assess or demonstrate clonal selection or SHM: a 3D human tonsil model incorporating primary FRCs, which reproduced all evaluated processes except SHM [12] (Figure 3G); B cells cocultured with CD40L‐expressing fibroblasts, which reproduced all processes except clonal selection [16]; and a microbead‐based CD40L stimulation platform, which also lacked clonal selection [17]. The most commonly used stimuli among these models included CD40L or anti‐CD40 [10, 11, 16, 17], IL‐4 [10, 11, 16, 17], IL‐21 [11, 16, 17], and BAFF [9, 11, 17]. Unlike the other models, Wagar et al. [9] and Braham et al. [12] did not employ CD40 stimulation (Figure 3B,G), likely because endogenous Tfh cells within tonsil mononuclear cells provided physiological CD40 signaling. Moreover, Wagar et al. [9] used a minimal stimulation strategy using only BAFF and antigen.

Antigenic stimulation was applied in four of these six studies [9–12] (Figure 3B,D,F,G), whereas one did not include either antigenic or anti‐BCR stimulation [16], and another used polyclonal stimulation with anti‐BCR and CpG [17]. Although it does not support SHM or memory formation, the study by Sanjuan Nandin et al. [18] is notable for its focus on antigen‐specific B cell activation, a feature rarely addressed in vitro. Using streptavidin nanoparticles conjugated to CpG and antigen, along with IL‐6 and IL‐15, they achieved targeted stimulation of memory B cells, offering an alternative strategy to overcome the challenge of matching antigen‐specific B and T cells. This approach allowed for targeted stimulation of memory B cells via antigen recognition, mimicking a critical early step of the GC response.

Most studies employed coculture systems to provide B cell costimulation. Zhong et al. [11] (Figure 3F) and Verstegen et al. [16] used CD40L‐expressing fibroblasts to deliver CD40 signaling, with Zhong et al. additionally adding tonsil‐derived FDCs. Braham et al. [12] cocultured human tonsil mononuclear cells with autologous or allogeneic FRCs in a 3D hydrogel (Figure 3G). Goyal et al. [10] cocultured B cells with autologous T cells and monocyte‐derived dendritic cells (Figure 3D), whereas Wagar et al. [9] relied on the self‐organization of tonsil mononuclear cells without introducing an additional stromal component (Figure 3B). These coculture strategies increasingly incorporate both immune and stromal cell populations to better reproduce the cellular interactions that regulate GC responses.

Studies by Verstegen et al. [16] and Wagar et al. [9] have provided novel insights into human immune responses. By comparing transcriptomes of in vitro differentiated B cells with ex vivo B cells and antibody‐secreting cells (ASCs), Verstegen et al. discovered a novel pre‐ASC population present in lymphoid tissues. They also identified a GC‐like population generated in vitro from human naïve B cells that may follow an alternative trajectory toward memory B cell differentiation. Wagar et al. revealed interindividual variability in their model and described the functional relevance of plasmacytoid dendritic cells and CD4+ T cells to the influenza response.

Among the most sophisticated approaches, microfluidic devices that recapitulate GC processes have also provided particularly valuable insights. Goyal et al. [10] developed an “organ‐on‐a‐chip” using peripheral blood B and T cells, along with autologous monocyte‐derived dendritic cells (Figure 3D). This model functionally responds to various immune stimuli, allowing evaluation of vaccine and adjuvant responses among individual donors. Similarly, Zhong et al. [11] created hydrogel‐based synthetic immune organoids from PBMCs and integrated them into a microfluidic organ‐on‐chip with a CXCL12 gradient (Figure 3F). They assessed the responses of immunocompromised patients and identified GC disorganization and poor vaccine responsiveness in diffuse large B cell lymphoma. The incorporation of CXCL12 into the model could enhance its physiological relevance. As a ligand for CXCR4, which is highly expressed in proliferating centroblasts, CXCL12 directs B cell migration and compartmentalization within the DZ of the GC [51]. Furthermore, CXCL12‐CXCR4 signaling is dynamically regulated during the B cell cycle, coordinating the movement of cells between dark and LZs to support iterative rounds of mutation and selection [52, 53]. These two models are particularly relevant for clinical and preclinical applications in vaccine testing and personalized immunology.

## 5. Murine Models Supporting the Study of GC Mechanisms

Naïve B cells were the most frequently used population in these studies [35–38, 43–45, 47]. Some models also demonstrated functionality with either naïve or GC B cells [39, 40, 42], whereas George et al. [41] specifically used GC B cells from Peyer′s patches, and Grund et al. [46] relied on memory B cells. Coculture strategies were common; nearly half of the studies included 40LB feeder cells [35, 38–40, 42, 45, 47]. This system, first established by Nojima et al. [38], consists of 3T3 fibroblasts engineered to express CD40L and secrete BAFF, mimicking T cell help. Other studies used cocultures with T cells [37, 43] or a combination of T cells and dendritic cells [41] (Table 2).

Five models were based on 3D cultures, related to the same research group and progressively improved with different biomaterial scaffolds, bioactive peptides, and stimulatory signals [35, 39, 40, 42, 45]. Purwada et al. [42] and Béguelin et al. [40] used an RGD‐presenting hydrogel reinforced with silicate nanoparticles, combined with IL‐4 and 40LB cells (Figure 4A). An intermediate study showed that modifying the hydrogel composition differentially regulated B cell activation and antibody production, further expanding the versatility of the platform [54]. Purwada et al. [39] subsequently transitioned to a maleimide (MAL)‐polyethylene glycol (PEG) hydrogel functionalized with REDV or RGD peptides, supplemented with IL‐4, 40LB cells, BCR stimulation, and anti‐Fas signaling (Figure 4B). Graney et al. [45] further adapted a MAL‐functionalized four‐armed PEG (PEG‐4MAL) hydrogel with REDV peptides, 40LB cells, an interleukin mix, and BCR stimulation (Figure 4C), whereas Moeller et al. [35] integrated the PEG‐4MAL/REDV system with 40LB cells, IL‐4, and candidate vaccines (Figure 4D).

**Figure 4:**
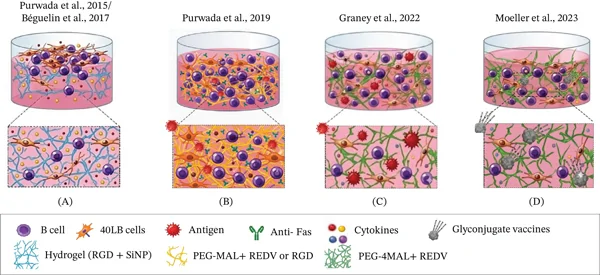
Schematic overview showing the evolution of 3D murine culture systems used to recreate GC‐like features. (A) Purwada et al., 2015 and Béguelin et al., 2017: RGD‐functionalized nanocomposite hydrogel with naïve B cells, 40LB cells, and cytokines. (B) Purwada et al., 2019: PEG‐MAL hydrogel functionalized with REDV or RGD peptides incorporating BCR stimulation and anti‐Fas signaling. (C) Graney et al., 2022: PEG‐4MAL/REDV hydrogel supporting antigen‐responsive naïve B cell–40LB cocultures. (D) Moeller et al., 2023: PEG‐4MAL/REDV hydrogel adapted as a preclinical vaccine screening platform.

### 5.1. Mouse Models Recapitulating Multiple GC Processes

Only two studies reproduced the full spectrum of GC processes searched: clonal selection, B cell proliferation, SHM, CSR, and differentiation into both plasma and memory B cells [35, 36], whereas another achieved all except SHM [37] (Table 2). These models employed naïve B cells; however, Martinez‐Riaño et al. [37] used 4‐hydroxy‐3‐nitrophenylacetyl (NP)‐specific B cells cocultured with ovalbumin (OVA)‐specific T cells and stimulated with NP‐OVA. This system allows the dissection of B:T cell interactions in an antigen‐specific context but does not represent true clonal selection, as the culture did not start with a polyclonal B cell population. In contrast, Moeller et al. [35] and Roh et al. [36] demonstrated clonal selection using glycoconjugate vaccine candidates or OVA‐ or NP‐coated microbeads, respectively.

Stimulation strategies varied among these models. Martinez‐Riaño et al. [37] relied on T cell help and antigenic stimulation, whereas Moeller et al. [35] and Roh et al. [36] included defined exogenous stimuli, such as IL‐4, BAFF, and CD40L, with Roh et al. additionally supplementing IL‐21. Moreover, Moeller et al. [35] created a 3D PEG‐4MAL hydrogel scaffold functionalized with REDV peptides to enhance cell–cell interaction (Figure 4D). These models show distinct strategies for reproducing multiple GC processes, from antigen‐specific cocultures to biomaterial‐supported systems with defined cytokine stimulation.

Among the main contributions, Moeller et al. [35] showed that their immune organoids can recapitulate GC‐like processes, including SHM and repertoire diversification in response to glycoconjugate vaccines. They observed robust BCR clustering and upregulation of key regulators of B cell differentiation and fate during the GC response (EZH2, IRF4, and BLIMP‐1). Despite the absence of T cells, this platform could be useful for prescreening vaccines and generating antigen‐specific antibodies, faster than traditional animal‐based immunization approaches. To better mimic the spatial and multivalent nature of cellular interactions in the GC, Roh et al. [36] developed a synthetic GC model with microbeads coated with CD40L and antigen, simulating Tfh‐ and FDC‐mediated stimulation. The use of surface‐bound, multivalent antigen allowed for more efficient B cell activation compared to soluble antigen. This system successfully promoted clonal enrichment and affinity‐dependent selection; however, the frequency of antigen‐specific B cells remained low, requiring additional enrichment by FACS to isolate antigen‐specific populations.

Martínez‐Riaño et al. [37] established a reductionist, antigen‐specific in vitro model based on two‐cell–type cocultures of naïve B and T cells. In this system, BCR‐mediated phagocytosis of particulate antigen promoted antigen presentation and the generation of high‐affinity, class‐switched immunoglobulins. This approach revealed that bystander B cells (those not engaged in cognate antigen recognition) failed to form stable T–B conjugates and instead preferentially differentiated into memory‐like cells. Altogether, the study demonstrates that BCR‐dependent antigen phagocytosis is a critical determinant of the quality of T cell help and subsequent B cell fate decisions, providing a valuable framework for ﻿studying affinity maturation and memory﻿‐like B cell formation in vitro.

The study by Kramer et al. (2022) is noteworthy for its innovative use of liposomes conjugated with anti‐IgM to mimic the fluid FDC membrane, although the system did not include antigenic stimulation to fully recapitulate a GC reaction. While the anti‐IgM does not represent a true antigen, this approach effectively simulated membrane‐bound BCR engagement, promoting more efficient BCR clustering and antigen internalization compared to soluble stimulation, which resulted in B cell proliferation, CSR, and plasma cell differentiation.

Together, these GC models highlight how engineering antigen presentation and multivalent stimulation can enhance B cell activation and selection, offering powerful tools to dissect GC‐like processes and accelerate antibody discovery.

## 6. Comparative Analysis of Human and Mouse In Vitro Models Recapitulating GC Processes

Both human and mouse in vitro systems have successfully replicated multiple GC processes, including clonal selection, B cell proliferation, SHM, CSR, and differentiation into plasma and memory B cells. To support these complex cellular events, coculture strategies are common, using CD40L‐expressing fibroblasts, T cells, or accessory stromal and dendritic cells to deliver essential costimulatory signals.

Human models showed greater diversity in cellular sources, including naïve and memory B cells, tonsil‐derived organoids, and PBMC‐based systems. Some of these models use T cells or FDCs, which naturally provide CD40 ligation and cytokine signals, respectively. In contrast, mouse models typically rely on naïve B cells and involve fewer variations in coculture configurations, with most experiments utilizing 40LB feeder cells to provide CD40L and BAFF signaling.

To mimic Tfh‐ and FDC‐derived stimuli, both human and mouse models integrate cytokines and costimulatory factors such as CD40L and IL‐4 as central drivers, often complemented by BAFF and IL‐21. Mouse systems generally use simpler stimulation schemes, whereas human models employ a broader repertoire of cytokines and costimulatory signals, including IL‐2, IL‐10, IL‐6, IL‐15, CpG, and CXCL12, replacing multiple physiological interactions.

CD40–CD40L engagement is essential for GC initiation, driving B cell proliferation, isotype switching, and affinity maturation [55]. IL‐4 from Tfh cells enhances B cell proliferation, CSR, and memory differentiation [56–58], whereas IL‐21 promotes clonal expansion, plasma cell generation, and affinity maturation [59–62]. Additional cytokines fine‐tune the GC environment: IL‐10 supports GC maintenance by promoting DZ phenotypes and proliferation through Tfr‐derived signals [63], though it can also favor extrafollicular plasma cell differentiation depending on context [64, 65]. BAFF, secreted by FDCs, contributes to GC B cell survival and follicular organization [66, 67]. Collectively, these components shape functional in vitro GC models that increasingly recapitulate human humoral immune responses.

To more closely mimic the GC microenvironment, some models developed 3D culture systems with synthetic hydrogels functionalized with adhesion peptides such as REDV and RGD. These bio‐adhesive motifs replicate essential interactions between GC B cells and components of the extracellular matrix (ECM) or stromal cells. Specifically, *α*v*β*3 integrins expressed by GC B cells bind to RGD‐containing domains of vitronectin present in the GC ECM [54, 68], whereas *α*4*β*1 integrins mediate adhesion both to vascular cell adhesion molecule‐1 (VCAM‐1) expressed on FDCs and the REDV‐containing domains of fibronectin [39, 68]. These ligand–integrin interactions support B cell adhesion, positioning, and activation within the GC [69, 70], emphasizing the importance of specific ligand–integrin pairings in regulating hydrogel‐mediated cellular responses [39].

Mouse systems offer some advantages, particularly in their capacity for genetic manipulation and antigen‐specific experimental design. These systems enable controlled investigation of defined B:T cell interactions, the use of transgenic antigen receptors, and the dissection of signaling pathways in a way that currently remains difficult to achieve in human models. This shows how mouse models provide superior experimental flexibility and control for mechanistic studies of the GC response.

However, murine models have inherent limitations when extrapolating to human immune responses, given interspecies differences in B cell repertoires (including differences in N‐region addition, germline gene usage, and complementarity‐determining region 3 of the immunoglobulin heavy chain [CDRH3] length in humans and mice) [71], distinct dynamics in transitional and follicular mature B cell populations across species [72], and divergent functional roles of cytokines such as IL‐10 [73]. In contrast, human systems capture greater cellular diversity and clinical relevance, offering a more physiologically relevant representation of human immune dynamics. Although experimental manipulation is more restricted in human systems compared to murine models, human‐based GC models offer greater translational potential for applications such as personalized immunotherapies, vaccine testing, or antibody‐based adoptive transfer strategies.

## 7. Conclusions and Future Perspectives

GC in vitro models have emerged as powerful tools for dissecting human adaptive immune responses. These systems have allowed the identification of novel immune cell populations present in lymphoid tissues [16], revealed interindividual variability in immune dynamics, and provided insights into the functional relevance of specific immune subsets in responses to pathogens such as influenza [9]. Furthermore, they offer a controlled platform for the evaluation of vaccine and adjuvant efficacy, enabling detailed assessment of immune responses, including in immunocompromised individuals [11].

Most in vitro models rely on polyclonal activation strategies, such as stimulation with anti‐BCR, CpG, or combinations of cytokines like IL‐4 and IL‐21. While these approaches successfully induce B cell proliferation and differentiation, they bypass antigen‐specific BCR engagement, which is fundamental to GC physiology. In vivo, the GC is defined by a highly selective process in which only B cells with sufficient affinity for a specific antigen are activated, expanded, and retained through iterative cycles of SHM and selection. This clonal selection is critical for developing high‐affinity antibodies and long‐lived memory B cells [1, 2]. Polyclonal activation, in contrast, drives a broad, nonspecific response that does not reflect the clonal selection dynamics present in a physiological GC, limiting the capacity of these systems to model antigen‐driven affinity maturation or study antigen‐specific B cell responses in detail.

Models incorporating defined antigens for BCR‐mediated activation could provide a more faithful representation of GC responses. These antigen‐specific systems enable selective activation of B cell clones, potentially mimicking clonal dynamics and competitive selection seen in vivo. The use of antigen‐coupled particles, multimeric antigens, or membrane‐bound antigens has been shown to improve B cell activation, promoting BCR clustering [74] and clonal expansion [18, 37, 44].

In vitro GC models offer significant advantages, including experimental control, cost‐effectiveness, reproducibility, and the ability to study specific mechanisms. However, their reduced complexity compared to in vivo systems limits representation of complete physiological responses. These models are particularly valuable for mechanistic studies to identify signals and molecules regulating GC formation and function. Additionally, they provide a platform with potential applications in vaccine design to generate more potent and long‐lasting immune responses and in the development of new immunotherapies targeting autoimmune diseases, cancer, and infectious diseases.

Future improvements for in vitro GC models should aim to refine clonal selection processes, ensuring antigen specificity rather than relying on polyclonal activation. A related consideration is the initial number of B cells used to start these cultures. Given that antigen‐specific precursors are typically present at extremely low frequencies in naïve repertoires, starting cultures with too few B cells may prevent the detection of specific responses, even under optimal stimulation conditions. Thus, future platforms must account for precursor frequency to ensure that antigen‐specific clones can emerge, expand, and be experimentally tracked.

Further advances will require improving the structural and functional complexity of GC‐like systems. Incorporating stromal cells or biomaterials that mimic lymphoid architecture may enhance physiological relevance, whereas reproducing the dynamic migration of B cells between light and DZs remains a major technical challenge. The integration of CRISPR‐Cas9 technology offers powerful opportunities to dissect gene‐specific functions. Additionally, optimizing fluid dynamics to simulate lymphatic flow could enhance nutrient and cytokine distribution. Finally, recreating oxygen gradients characteristic of different GC zones, where hypoxic microenvironments may influence B cell metabolism and selection, represents a promising but underexplored area to advance the fidelity of GC‐like environments in vitro.

## Author Contributions

**Rocio Quinto-Manzanares:** conceptualization, investigation, visualization; **Alondra Yareli Martínez-Mora:** investigation, visualization; **Velia Verónica Rangel-Ramírez:** writing – review and editing; **Jesús Martínez-Barnetche**: writing – review and editing; **Hilda Minerva González-Sánchez:** conceptualization, writing – original draft, review and editing, funding acquisition.

## Funding

This study was supported by Secretaría de Ciencia, Humanidades, Tecnología e Innovación, CF‐2023‐I‐2387.

## Conflicts of Interest

The authors declare no conflicts of interest.

## Supporting Information

Additional supporting information can be found online in the Supporting Information section.

## Supporting information


**Supporting Information 1** Table S1. Summary of studies that analyze or report on aspects related to germinal center (GC) formation, immunological memory, and antibody (Ab) production using human B cells in vitro.

**Supporting Information 2** Table S2. Summary of studies that analyze or report on aspects related to germinal center (GC) formation, immunological memory, and antibody (Ab) production using murine B cells in vitro.

**Supporting Information 3** Table S3. Summary of human 3D in vitro germinal center models.

## Data Availability

Data sharing not applicable to this article as no datasets were generated or analyzed during the current study.
