# Supplementary material for: Advances in Human and Animal Germinal Center Models In Vitro: New Approaches to Studying Immune Responses
Source: Biomed Res Int. 2026 Oct 5;2026:2510652. doi: 10.1155/bmri/2510652 (PMC13636671; doi:10.1155/bmri/2510652)
Supplement: Supplementary file 1 [file BMRI-2026-2510652-s001.docx]

| Reference | GC | Immune  Memory | Abs production | Comments |
| --- | --- | --- | --- | --- |
| Arpin C, et al., 1995 | ***** | ***** | ***** | Robust model that generates memory and plasma cells from germinal center B cells *in vitro*. |
| Arpin C, et al., 1997 | ***** | ***** | ***** | Explores how memory B cells differentiate more easily into plasma cells; useful for both processes. |
| Choe J & Choi YS, 1998 | ***** | ***** | ***** | Studies how IL-10 promotes plasma cell differentiation rather than memory expansion. |
| Wagar LE, et al., 2021 | ***** | ***** | ***** | Organoids that recreate GC dynamics, memory, and antibody production *ex vivo*. |
| Braham MVJ, et al., 2023 | ***** | ***** | ***** | 3D model that enhances survival, memory, and functional differentiation. |
| Goyal G, et al., 2022 | ***** | ***** | ***** | Organ-on-a-chip that recreates follicles, memory, and humoral response to vaccines. |
| Helm M, et al., 2023 | ***** | ***** | ***** | First steps toward encapsulated artificial GC; model for immunological memory and production of antibodies. |
| Verstegen NJM, et al., 2023 | ***** | ***** | ***** | Single-cell profile of differentiation, memory, and antibody secretion. |
| Kuchen S, et al., 2007 | ***** | ***** | ***** | IL-21+CD40L signaling is central to activation, expansion, GC, and antibody production. |
| Shaw J, et al., 2010 | ***** | ***** | ***** | Studies pDC–B cell interaction via CD70/CD27, implicated in memory, GC, and antibody production. |
| Zhong Z, et al., 2025 | ***** | ***** | ***** | Human organoids that recapitulate B-T interaction and antibody production; useful for studying response to lymphoma. |
| Braham MVJ, et.al. 2026 | * | * | * | 3D human tonsil culture supporting antigen-specific B-cell activation, GC-like responses and antibody production. |
| Galibert L, et al., 1994 | ***** | ***** |  | Prolonged GC-type activation model induced by CD40 + IL-4; proliferation is maintained. |
| Choe J, et al., 1997 | ***** | ***** |  | Focus on IL-4 + BCR for the generation of memory cells from GCs. |
| Kuo TC, et al., 2007 | ***** | ***** |  | Identifies the role of BCL-6 in differentiation into memory cells from a GC-like context. |
| Schmidt A & Baumjohann D, 2022 | ***** | ***** |  | *Ex vivo* tonsillar microenvironment useful for studying GCs and Tfh-B memory. |
| Dubois B, et al., 1999 | ***** |  | ***** | Studies how dendritic cells induce proliferation and isotype switching. |
| Maarof G, et al., 2010 | ***** |  | ***** | Shows how IL-24 inhibits plasma cell differentiation from GC cells. |
| Ding BB, et al., 2013 | ***** |  | ***** | IL-21 + CD40L induce Blimp-1 expression, key in plasma differentiation. |
| Vijayan D, et al., 2016 | ***** |  | ***** | IL-27 promotes the differentiation of naïve B cells into a GC B cell-like phenotype. |
| Koers J, et al., 2022 | ***** |  | ***** | Studies how hypoxia regulates GC-like maturation and IgG isotype switching. |
| Grimaitre M, et al., 1997 | ***** |  | ***** | *In vitro* model recapitulates the GC-like phase (CD38^int^, CD77⁺), followed by differentiation into plasma cells. |
| Suthanthiraraj PPA, et. al. 2025 | * |  | * | Synthetic microbead niches promote naïve B-cell expansion, differentiation and antibody secretion. |
| Galibert L, et al., 1996 | ***** |  |  | Focuses on inducing GC phenotypic characteristics through CD40 and BCR signaling. |
| Choe J, et al., 1996 | ***** |  |  | Studies the regulation of apoptosis in GC B cells stimulated with CD40L and anti-Ig. |
| Pound JD & Gordon J, 1997 | ***** |  |  | Focuses on maintaining the viability/phenotype of GC B cells *in vitro*. |
| Pound JD, et al., 1999 | ***** |  |  | Focuses on CD40 signaling and minimal requirements to prevent apoptosis. |
| Choe J, et al., 2000 | ***** |  |  | Study of cellular interactions in the GC microenvironment using a cell line. |
| Llibre A, et al., 2016 | ***** |  |  | Studies activation and migration markers in human GC. |
| Schmidt A, et al., 2020 | ***** |  |  | *Ex vivo* culture preserves the human GC microenvironment for pharmacological studies. |
| Kim J, et al., 2013 | ***** |  |  | Use of human FDCs to maintain GC B cells; focuses on survival signaling. |
| Fenner RE, et.al. 2025 | * |  |  | Long-term culture and genetic manipulation of human GC B cells to model germinal center biology and lymphomagenesis. |
| Morrison AI, et.al. 2025 | * |  |  | Organotypic human lymph node model supporting immune cell interactions and GC-like microenvironments. |
| Havey L, et.al. 2025 | * |  |  | Hypoxic GC-like model of EBV-transformed B cells reveals metabolic requirements during GC-like growth. |
| Ettinger R, et al., 2005 |  | ***** | ***** | Evaluates the effect of IL-21 on naive and memory B cells toward plasma differentiation. |
| Good KL, et al., 2006 |  | ***** | ***** | Analyzes IL-21 as a stimulant of expansion and differentiation of naive and memory B cells. |
| Kwakkenbos MJ, et al., 2010 |  | ***** | ***** | Uses genetic engineering to expand antibody-producing memory B cells. |
| Sanjuan Nandin I, et al., 2017 |  | ***** | ***** | Model for rapidly generating specific Ab-producing B cells from memory. |
| Jourdan M, et al., 2019 |  | ***** | ***** | Model of terminal differentiation of memory B cells into long-lived plasma cells. |
| Fecteau JF, et al., 2009 |  | ***** | ***** | Focuses on memory B cells and antibody secretion after CD40 stimulation. |
| Vásquez C, et al., 2015 |  | ***** | ***** | Studies circulating IgM memory B cells, which proliferate and differentiate into IgM-secreting cells upon stimulation. |
| Silva JM, et.al. 2025 |  | * | * | Expansion of antigen-specific memory B cells from PBMCs for monoclonal antibody recovery. |
| Conde L, et.al. 2026 |  | * | * | Single-cell B-cell culture platform for memory B-cell expansion and antibody discovery. |
| Good KL, et al., 2009 |  | ***** |  | Characterizes functional properties of memory B cells. |
| Su KY, et al., 2016 |  | ***** |  | Studies the capacity of naive and memory B cells as antigen-presenting cells. |
| Unger PA, et al., 2021 |  |  | ***** | Focuses on differentiation of naive B cells into plasmablasts without a GC microenvironment. |
| Pignarre A, et al., 2021 |  |  | ***** | Analyzes cellular steps and signaling toward plasmablasts, without a GC model. |
| Dubois B, et al, 1998 |  |  | ***** | IL-12 promotes the differentiation of naive B cells into IgM⁺ plasma cells; study with human DCs. |
| Huggins J, et al., 2007 |  |  | ***** | Focuses on naive CD27⁻ cells, but compares it with memory cells; plasma cells are generated. |
| Haque S, Diamond B. 2025 |  |  | * | Primary human B-cell culture showing reduced plasma cell differentiation after myricetin treatment. |
| Schlotheuber LJ, et. al. 2026 |  |  | * | Functional analysis of activated human B cells with heterogeneous antibody secretion profiles. |

**Table S1.** Summary of studies that analyze or report on aspects related to germinal center (GC) formation, immunological memory, and antibody (Ab) production using human B cells *in vitro*. Relevant observations or methodological specifics are also noted in the comments. Abbreviations: BCL-6, B cell lymphoma 6; BCR, B cell Receptor; BLIMP-1, B-Lymphocyte- Induced Maturation Protein 1; DCs, Denditric cells; FDCs, Follicular Dendritic Cells; GC, Germinal center; IgG, Immunoglobulin G; IL-, Interleukin-; pDC, Plasmacytoid dendritic cell; Tfh, T follicular helper cells.
