# Supplementary material for: Advances in Human and Animal Germinal Center Models In Vitro: New Approaches to Studying Immune Responses
Source: Biomed Res Int. 2026 Oct 5;2026:2510652. doi: 10.1155/bmri/2510652 (PMC13636671; doi:10.1155/bmri/2510652)
Supplement: Supplementary file 2 [file BMRI-2026-2510652-s002.docx]

| Reference | GC | Immune  Memory | Abs production | Comments |
| --- | --- | --- | --- | --- |
| Kosco MH, et al., 2022 | * | * | * | Functional model that simulates the germinal microenvironment. |
| George A, et al., 1991 | * | * | * | Isolates unique GC clones and measures their function. |
| Kosco MH, et al., 1988 | * | * | * | Analyzes functional interactions in mixed culture. |
| Tew JG, et al., 1995 | * | Partial | * | Confirms the trophic role of FDC for GCs. |
| Kotloff DB, et al., 1988 | Partial | Partial | * | Models T–B clone microinteractions. |
| Klinman NR, 1996. | Partial | * | * | Promotes expansion of memory clones. |
| Dahlenborg K, et al., 1997 | * | Partial | * | Helps understand the terminal transition of human GC cells. |
| Jin H & Malek TR, 2006 | * | * | * | Elucidates how IL-4 and IL-21 maintain the germinal phenotype in activated B cells. |
| Nojima T, et al., 2011 | * | * | * | *In vitro* platform that mimics the GC and directs B cell fate toward memory or plasma cells. |
| Purwada A, et al., 2015 | * | * | * | Functional 3D organoid with T cell and FDC compartments that simulates GC reactions. |
| Abbott RK, et al., 2016 | * | * | * | Impact of hypoxia on optimizing GC responses. |
| Purwada A & Singh A, 2017 | * | * | * | Allows modulation of signals that control antibody development and secretion by B cells. |
| Haniuda K, et al., 2017 | * | * | * | Platform for producing mature B cells *in vitro*. |
| Chu VT, et al., 2017 | * | * | * | Tool to modify GC B cells and observe functional effects. |
| Roh KH, et al., 2018 | * | * | * | Demonstrates that defined signals are sufficient to induce humoral immunity without the need for stroma. |
| Purwada A, et al., 2019 | * | * | * | Organoids that allow the study of GC functional development. |
| Robinson MJ, et al., 2019 | * | * | Partial | Defines the difference between signals for survival vs. differentiation. |
| Lopez-Ocasio M, et al., 2020 | * | Partial | * | Useful model for studying memory suppression vs. active response. |
| Graney PL, et al., 2020. | * | * | * | Reveals epigenetic regulation in age-dependent GC responses. |
| Arita K, et al., 2013 | * | * | * | Retroviral model that reproduces GCs *in vitro*; useful for analyzing memory, antibodies, and GCs. |
| Moeller et al., 2023 | * | * | * | Organoids evaluated with conjugate vaccines; they recapitulate GC, memory, and antibody secretion |
| Martínez-Riaño A, et al., 2023 | * | * | * | Functional GC system with only B+T and antigenic particle; phagocytosis mechanism favors selection and SHM. |
| Graney PL, et al. 2022 | Partial | * | Partial | Organoids that mimic early memory signals; model part of the GC *ex vivo*. |
| Lanahan SM, et al., 2024 | Partial | * | * | Links PI3Kγ-mediated intracellular signaling with efficient Ab production. |
| Kim Y, et al., 2024 | Partial | * | * | Defines how IL-21 modulates B responses depending on the context, reinforcing its key role in antigen activation. |
| Koike T, et al., 2019 | Partial | * | Partial | Shows how different CD40 signal intensities direct memory B cell subpopulations. |
| Vijayashankar DP, Vaidya T 2022 | Partial | Partial | Partial | Delays differentiation, favors cell fate diversification; applies to memory and GCs. |
| Beagley KW, et al., 1991 | * | * |  | IL-6 can simulate rapid memory differentiation. |
| Lahvis GP & Cerny J, 1997 | * | * |  | Reproduces CD40L + BCR cross-linking signals from GCs. |
| Aydar Y, et al., 2005 | * | Partial |  | Complexed FDCs facilitate maturation and isotype switching. |
| Fisher JS, et al., 2024 | * | * |  | Explains how the aging microenvironment affects Tfh function and GC formation. |
| Kim SJ, et al., 2008 | Partial |  | * | IL-12 changes fate towards plasmablasts to the detriment of GC. |
| Purwada A, et al., 2017 | * |  | * | Modular protected organoids; GC activation, secretion, and phenotype are promoted *ex vivo*. |
| Kramer L, et al., 2022 | * |  | Partial | Synthetic system that reproduces antigen presentation in “mini-GCs”; useful for analyzing GCs. |
| Béguelin W, et al., 2017 | * |  | Partial | Highlights the epigenetic role of EZH2 in GC formation; less focus on antibodies/memory. |
| Wu Y, et al., 2008 | * |  | Partial | Evidence of late antigenic signals from FDCs that promote SHM in the GC. |
| Morafo V, et al., 1999 | * |  |  | Simplified model that reproduces Th2 cell signals *in vitro* to study GC induction. |
| Knödel M, et al., 2001 | Partial |  |  | Genetic approach allows us to study how Blimp-1 overexpression drives terminal B cell differentiation despite blocking isotype switching. |
| Lebman DA & Coffman RL, 1988 |  | * | * | Functional differences in mucosa according to cytokines. |
| Nishikawa S, et al., 1980 |  | * | * | Stimulates adaptive memory *in vitro*. |
| Shapiro SM, et al., 2003 |  | * | * | Confirms the role of Blimp-1 in the molecular regulation of B cell differentiation into plasma cells. |
| Bhattacharya D, et al., 2007 | Partial | * | * | Transcriptomic profile reveals differentiation pathways toward memory after antigenic stimulation. |
| Pihlgren M, et al., 2013 |  | * | * | Novel model of T cell-independent class switching using peptide liposomes. |
| Inagaki T, et al., 2013 |  | * | * | Alternative system for inducing T cell-independent class switching with murine splenocytes *in vitro*. |
| Brewer MG, et al., 2017 |  | * | * | Immunogen design to better target B cell responses. |
| Grund LZ, et al., 2013 |  | Partial | * | Shows generation of long-lived plasma cells with IL-17A signals. |
| Wysocki LJ, et al., 1992 |  |  | * | Protocol that allows class change without somatic mutation or germinal center formation. |
| Taguchi H, et al., 1990 |  |  | * | CD5– B cells produce IgG after stimulation with LPS and immune complexes *in vitro*. |
| Wilson BS et al., 1985 |  |  | * | Explores the CR2 receptor and its mitogenic role. |
| Robinson MJ &Tarlinton DM, 2025 |  |  | * | IL-21 drives plasmablast differentiation and immunoglobulin secretion *in vitro.* |
| Pone et al., 2022 |  |  | * | Study isotype switching; key to antibody production. |

**Table S2.** Summary of studies that analyze or report on aspects related to germinal center (GC) formation, immunological memory, and antibody (Ab) production using murine B cells *in vitro*. Relevant observations or methodological specifics are also noted in the comments column. Abbreviations: BCR, B cell Receptor; BLIMP-1, B-Lymphocyte- Induced Maturation Protein 1; EZH2, Enhancer of Zeste Homolog 2; FDCs, Follicular Dendritic Cells; IL-, Interleukin-; LPS, Lipopolysaccharide; PI3Kγ, phosphoinositide 3-kinase γ; Tfh, T follicular helper cells; Th2, T helper 2 cells; SHM, Somatic Hypermutation.
