# Supplementary material for: Advances in Human and Animal Germinal Center Models In Vitro: New Approaches to Studying Immune Responses
Source: Biomed Res Int. 2026 Oct 5;2026:2510652. doi: 10.1155/bmri/2510652 (PMC13636671; doi:10.1155/bmri/2510652)
Supplement: Supplementary file 3 [file BMRI-2026-2510652-s003.docx]

| **Reference** | **Culture Model** | **Stimuli** | **Cellular Components** | **Findings** | **Limitations** |
| --- | --- | --- | --- | --- | --- |
| Zhong Z, et al, 2025 | Human PBMC organoids encapsulated in 3D synthetic hydrogels (7.5% w/v PEG 4MAL) conjugated with adhesive peptides RGD and GFOGER, chemically crosslinked with VPM and DTT. Integration in an organ-on-a-chip system. | Day 0-2: IL-4 and BAFF; Day 2-final (day 12): IL-21. Inactivated influenza A virus or Fluzone antigen ± R848 adjuvant. CXCL12. | Total PBMCs or tonsil mononuclear cells, CD40L-expressing fibroblasts, human-tonsil-derived FDCs. | PBMCs organoids maintain GC B cells and plasma  cells longer than tonsil-derived ones, exhibiting SHM and CSR. Addition of CXCL12 allowed polarized distribution of cell populations. Supports long-term culture (>2 weeks). | Technically complex (Microfabrication, Microfluidic control, Specialized monitoring); difficult to access; difficult to introduce genetic tools. |
| Goyal et al., 2022 | "Organ-on-a-Chip" microfluidic device with two parallel channels separated by a porous membrane. Cells were suspended in a solution of Matrigel and collagen I and introduced into the chip's lower channel. After gelation, the upper channel was filled with medium, simulating blood flow. | Culture perfused with activators such as SAC, IL-4, anti-CD40 or an influenza vaccine. | Human B and T cells derived from peripheral blood, supplemented with autologous monocyte-derived DC. | B cells expressed AID and displayed plasma differentiation. They replicated donor-specific responses to the vaccine, with plasmablast formation and IgG secretion. Microfluidic flow prevents spontaneous activation, ensuring model control. | Technically complex (Microfabrication, Microfluidic control, Specialized monitoring); difficult to access; difficult to introduce genetic tools. |
| Wagar et al., 2021 | Human tonsil tissue, mechanically and enzymatically dissociated, cultured on low-adherence plates where cell aggregates were reconstituted by self-regrouping to high density. | BAFF and antigens (LAIV, WT influenza virus, MMR vaccine, R-PE, rabies vaccine, or the Ad5-vectored SARS-CoV-2 candidate vaccine). Alum or imiquimod were tested as adjuvants. | B cells, T cells, macrophages, DC and stromal cells, all from the tissue, without prior selection. | Production of antigen-specific antibodies, SHM and affinity maturation, plasmablast differentiation and CSR. | Dependent on human tissue availability, variability in cellular composition, difficult scalability for high-throughput screening. |
| Braham et al., 2023 | Synthetic PEG-4MAL hydrogel with 5% polymer and 2 mM RGD peptide. | Multiple stimulation protocols. The selected cytokines mixture contained: CpG, IL-2, IL-4, IL-10, IL-10, IL-21, IL-6, IFNα, and ARPIL. | B cells, tonsil-derived dermal stromal cells, and irradiated CD40L-expressing fibroblasts. | High survival and cell proliferation of B cells, differentiation into plasmablasts and plasma cells, production of IgG/IgA. | There was no direct BCR stimulation or addition of antigen. Primary lymphoid stromal cells were derived from an allogeneic source. |
| Braham et al., 2026 | Human tonsil culture embedded in a 3D PEG-4MAL hydrogel functionalized with RGD | Recombinant SARS-CoV-2 and influenza antigens, vaccines, and inactivated influenza virus ± R848. | Human tonsil mononuclear cells co-cultured with autologous or allogeneic FRCs. | Increased GC-like B cells, antibody-secreting cells, antigen-specific B-cell frequencies and antigen-specific IgG production, and better preserved Tfh and other lymphocyte populations. | Requires fresh tonsil tissue and FRCs; technically demanding; donor variability; no evidence of de novo SHM or antigen-driven clonal selection. |
| Helm et al., 2023 | Microcapsules based on SCS and PDADMAC. | IL-4, IL-21, BAFF, and CD40L were added to the SCS solution used to suspend the cells. | Human tonsillar B cells. | Compared with suspension cultures, they induced a GC-like phenotype more rapidly and better sustained plasma cell survival. Supports long-term culture (>2 weeks). | There was no direct BCR stimulation or antigen addition; no other cell types were included. |

**Table S3.** **Summary of human 3D *in vitro* germinal center models.** The findings summarized correspond to key results that are either directly relevant to the GC model or were selected for their significance within the context of the culture system or the specific experimental approaches employed. Limitations were either reported by the authors or inferred based on the experimental design. Abbreviations; AID, Activation- Induced Cytidine Deaminase; APRIL, A Proliferation- Inducing Ligand; BAFF, B-cell Activating Factor; CD40L, CD40 Ligand; CpG, Cytosine-phosphate-Guanine; CSR, Class Switch Recombination; CXCL2, C-X-C motif chemokine ligand 2; DCs, Dendritic Cells; DTT, Dithiothreitol; FDCs, Follicular Dendritic Cells; FRCs, fibroblastic reticular cells; GC, Germinal Center; GFOGER, Gly-Phe-O-Gly-Glu-Arg where O = hydroxyproline; IFNα, Interferon-alpha; IL-, Interleukin-; LAIV, Live Attenuated Influenza Vaccine; MMR Vaccine, Measles, Mumps, and Rubella Vaccines; PBMCs, Peripheral Blood Mononuclear Cells; PDADMAC, poly-diallyl-dimethyl-ammonium chloride; PEG-4MAL, Poly(ethylene glycol)-4 arm- Maleimide; RGD, Arg-Gly-Asp; R-PE, R-phycoerythrin; SAC, *S. aureus* Cowan I antigen; SCS, Sodium Cellulose Sulfate; SHM, Somatic Hypermutation, VPM, Protease-sensitive peptide crosslinker (GCRDVPMS↓MRGGDRCG).
